# Imported malaria among people who travel to visit friends and relatives: is current UK policy effective or does it need a strategic change?

**DOI:** 10.1186/s12936-015-0666-7

**Published:** 2015-04-09

**Authors:** Ron H Behrens, Penny E Neave, Caroline OH Jones

**Affiliations:** Department of Clinical Research, Faculty of Infectious and Tropical Diseases, London School of Hygiene and Tropical Medicine, London, UK; Department of Public Health, Auckland University of Technology, Auckland, New Zealand; Department of Disease Control, Faculty of Infectious and Tropical Diseases, London School of Hygiene and Tropical Medicine, London, UK; Kemri-Wellcome Trust Research Programme, Kilifi, Kenya; Nuffield Department of Clinical Medicine, Centre for Tropical Medicine, University of Oxford, Oxford, UK

## Abstract

**Background:**

The proportion of all imported malaria reported in travellers visiting friends and relatives (VFRs) in the UK has increased over the past decade and the proportion of *Plasmodium falciparum* malaria affecting this group has remained above 80% during that period. The epidemiological data suggest that the strategies employed in the UK to prevent imported malaria have been ineffective for VFRs. This paper attempts to identify possible reasons for the failure of the malaria prevention strategy among VFRs and suggest potential alternatives.

**Methods:**

A review of the current UK malaria prevention guidelines was undertaken and their approach was compared to the few data that are available on malaria perceptions and practices among VFRs.

**Results:**

The current UK malaria prevention guidelines focus on educating travellers and health professionals using messages based on the personal threat of malaria and promoting the benefits of avoiding disease through the use of chemoprophylaxis. While malaria morbidity disproportionately affects VFRs, the mortality rates from malaria in VFRs is eight times, and severe disease eight times lower than in tourist and business travellers. Recent research into VFR malaria perceptions and practices has highlighted the complex socio-ecological context within which VFRs make their decisions about malaria. These data suggest that alternative strategies that move beyond a knowledge-deficit approach are required to address the burden of malaria in VFRs.

**Discussion:**

Potential alternative strategies include the use of standby emergency-treatment (SBET) for the management of fevers with an anti-malarial provided pre-travel, the provision of rapid diagnostic testing and treatment regimen based in general-practitioner surgeries, and urgent and walk-in care centres and local accident and emergency (A&E) departments to provide immediate diagnosis and accessible ambulatory treatment for malaria patients. This latter approach would potentially address some of the practical barriers to reducing the burden of malaria in VFRs by moving the process nearer to the community.

## Background

Between 2002 and 2013 there was a total of 17,811 reports of imported malaria in the UK [1]. The majority of cases were among individuals travelling to West African countries who had acquired *Plasmodium falciparum* whilst visiting friends and relatives (VFRs). For example, in 2013, the year for which the most recent data are available, 65% of all 1,501 malaria cases reported were acquired in West Africa, nearly 80% were caused by *P. falciparum* and 82% were among people who were VFRs [1].

The profile of imported malaria is similar in other European countries. In France between 2001 and 2004, of the estimated 6,500 to 7,000 annual cases, 83% were of *P. falciparum* and more than 90% of *P. falciparum* cases were imported from sub-Saharan Africa (SSA), the majority from West African countries [2]. More recent data from 2012 show that 95.8% of the estimated 3,510 cases imported into France were acquired in African countries, and 88% of infections were caused by *P. falciparum* [2,3]. In Italy, between 2000 and 2006, 83% of all 5,219 reports of imported malaria were caused by *P. falciparum* and the majority (93%) were acquired in sub-Saharan Africa [4].

**Figure 1:**
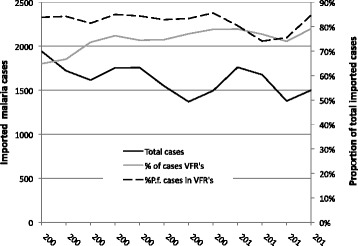
**The total UK imported malaria cases annually between 2002–2013, with the proportion of all malaria and** ***Plasmodium falciparum*** **cases reported in VFR travellers**. (Where reason for travel is known). Data provided by the PHE Malaria Reference Laboratory.

It is clear that imported malaria morbidity disproportionately affects VFRs. The proportion of all reported malaria cases in the UK affecting VFRs increased from 65% in 2002 to 79% in 2013, with the total proportion remaining above 70% and *P. falciparum* over 80% over the past ten years (Figure 1). In France a similar trend is seen, with the proportion of *P. falciparum* cases in VFRs increasing from 50% in 1996 to 82% in 2011 [3].

Despite the burden of morbidity, the case fatality rate from malaria among VFRs is significantly lower than that among non-VFRs. The data published from the UK Malaria Reference Laboratory (MRL) found that the case fatality was 3.0% in tourists compared with 0.32% in VFR travellers, an odds ratio of 8.2 [5]. Similar findings on the severity of malaria were found in France, where a significantly lower odds ratio of 0.25 of VFRs developing severe malaria compared to matched cases in travellers of European origin was reported [6]. These differences in case fatality rate between VFRs and non-VFRs with malaria and the potential role of immunity to malaria needs further research.

These data suggest that the malaria prevention policies in these two countries are failing to effectively target prevention of imported *P. falciparum* in VFR travellers who have a high incidence of infections, but low case fatality rates.

In light of this epidemiological evidence, the question was raised – are the current approaches to preventing malaria among VFRs in the UK the most appropriate way of dealing with the continuing burden of disease in VFRs, or should policy makers be rethinking the prevention strategy?

### UK malaria prevention policy: ABCD

In the UK the national recommended guidelines for malaria prevention are developed for Public Health England (PHE) by the Advisory Committee on Malaria Prevention (ACMP), a multi-professional group who formulate decisions based on a risk assessment of contracting malaria in country-specific destinations [7]. The Committee produces annual guidelines on malaria prevention for use by both healthcare workers who advise travellers, and for prospective travellers who wish to understand the malaria risks and options for mitigating them. The guidelines are based on a range of sources of information including: expert opinion, published research and the recommendations of other technical bodies, including the World Health Organization, the Centers for Disease Control USA, and the Malaria Atlas Project (Chiodini, pers comm). The acronym ‘ABCD’ reflects the main strategies in the UK guidelines, where A is an awareness of the risk, B refers to bite prevention, C stipulates the need for chemoprophylaxis, and D emphasises the importance of rapid diagnosis and treatment [7]. Within the guidelines, VFRs are recognized as a ‘special category’ but the advice proposed for them is similar to the advice set out for other travellers, particularly the requirement for chemoprophylaxis. There is, however, an added emphasis on the need to raise awareness in this high-risk group, emphasizing that malaria is not a trivial disease and that acquired semi-immunity may be quickly lost. The guidelines suggest that the best strategy for increasing the use of effective malaria prevention measures in VFRs is the dissemination of appropriately tailored health information targeted to migrant communities, especially of West African descent, stressing the importance of chemoprophylaxis use when travelling. The guidelines further state that health advisers for this group, including primary care practitioners working in areas with large numbers of migrants, can have an important role to play.

### VFR awareness of malaria risk (A)

Studies on the knowledge and perception of the risk of malaria among VFRs in the UK suggest that the majority are aware of the potential risk of contracting the disease when they travel to their country of origin [8,9] and similar knowledge levels have been found among VFR travellers living in France [10]. However, knowledge of malaria, its presence and transmission do not always mean that malaria is perceived as a significant health threat to be actively avoided [11-14].

The few data that are available on VFR perceptions of personal risk of severe illness and death from malaria suggest that their perceptions are at variance with the advice provided by PHE. For example, there are the frequent reports amongst VFR travellers of feelings of competence in their ability to self-manage the disease, both recognizing and managing symptoms of malaria [11]. Several studies have found that VFR travellers who suspected they had malaria while in their travel destination followed local practices and obtained treatment drugs from local providers (pharmacies, stores and street vendors) without a parasitological diagnosis, with many reportedly making a full recovery [11,12,15]. It is likely that these self-treatment behaviours were influenced by the context within which the disease was experienced, where the support of family and friends was available and the actions reflect common management practices in endemic destinations [16]. Recent research among a small group of VFRs in London suggests that among this group there was a perception, based on experience, that the risk of malaria was not necessarily life threatening to themselves, but could become a medical emergency if not treated in a timely and appropriate manner; that some groups of people (e.g., young children) were more likely to become severely ill if they contract the disease [12]. The study also found that a number of these VFRs had confidence in their ability, and that of doctors and pharmacists in their country of origin, to diagnose and treat malaria appropriately, but less confidence in the approach of practitioners in the UK National Health Service (NHS) to the identification and treatment of the disease [12]. Interestingly, these sentiments were echoed by some of the NHS health care providers interviewed as part of the same study [17], and similar findings have been reported from a study in the USA which investigated travel health practices of the Nigerian community living in Houston, USA [18].

A recent study of malaria mortality among VFRs in the UK argued that immunity following previous exposure was unlikely to be the only explanation of reduced severity, as the analysis included travellers born in the UK (second- or third-generation migrants) who would not have naturally acquired immunity. One explanation proposed was that VFRs may have a better understanding of malaria and its dangers, and may recognize the symptoms early and self-treat or obtain medical treatment quicker than other types of travellers [5]. This explanation corresponds with the data with the qualitative research findings on the perceptions and practices of VFRs [11,12,14,15].

### VFR use of bite prevention (B) and chemoprophylaxis (C)

While the data suggest that the behaviours and perceptions of many VFRs differ to the advice provided by PHE both in terms of their actions and their perceptions of the potential consequences should they contract malaria, there is evidence that many VFRs have a very good knowledge of the causes of malaria and the need to avoid being bitten by mosquitoes to prevent transmission [11,18]. The ACMP advice on bite avoidance highlights the use of window and door screens, insect repellents, spraying bedrooms with insecticide, and sleeping under bed nets [7]. Very few data are available detailing VFRs’ compliance with personal protective measures, but a recent qualitative study suggests that while all participants were aware of the need to avoid mosquito bites and most relied on their hosts to provide accommodation with adequately screened bedrooms, very few were willing to sleep under an insecticide-treated bed net [12].

Several studies have found that many VFRs are also frequently unlikely to comply with PHE advice on the use of chemoprophylaxis and, in general, chemoprophylaxis use among VFRs has been found to be considerably lower than among other travellers to malaria-endemic destinations. In a study assessing chemoprophylaxis use between 1999 and 2006 among travellers diagnosed with malaria on return from sub-Saharan Africa, only 7% of VFR travellers reported having used recommended drugs, compared with 24% of people travelling for other reasons [19]. Compliance to chemoprophylaxis recorded in malaria cases are not reliable estimates or representative of travellers practices, so airport surveys of departing passengers are a better snapshot of prophylaxis use. Departing UK passengers travelling to Nigeria and Ghana, of whom three-quarters were travelling as VFRs to each country, were found to have different compliance rates, with 50% of Nigerians and 82% of Ghanaian travellers reporting that they were taking chemoprophylaxis [8]. French data on imported malaria is not categorized by reason for travel, but where information is available, over 70% of VFR malaria cases had not used chemoprophylaxis [3]. In a study undertaken in 559 Dutch VFR malaria patients, 86% were similarly found to be poorly or non-compliant [20].

In the few data that are available, no association has been found between knowledge among VFRs of the risk of malaria and the use of chemoprophylaxis. Despite an accurate understanding of the threat of malaria among departing Dutch VFR travellers at high risk of malaria (74%), 27% of them reported no intention of undertaking risk-avoidance measures [21]. In a separate departure-lounge survey in The Netherlands, just over half of the high-risk VFR travellers (54%) used chemoprophylaxis despite an accurate perception of the threat of malaria [14].

Several authors have suggested that the cost of purchasing malaria chemoprophylaxis is an important barrier to the wider use of medication by VFR travellers in the UK, especially for those travelling as a family group, and/or compared to the low cost of purchasing malaria treatment should symptoms arise. Using modelling, some studies have suggested that an improved uptake of chemoprophylaxis would occur if their cost was subsidized by health systems [22,23], but an analysis of subsidized prophylaxis in the UK found a very marginal benefit in the impact of imported malaria in VFRs in a borough where subsidized anti-malaria drugs were available [24].

Difficulties in accessing chemoprophylaxis have also been reported as a constraint to its use particularly among VFRs who travel at short notice [10,12], and a study among VFRs of Southeast Asian origin found that a belief in their malaria immunity restricted the uptake of prophylaxis in this group [15]. While no single factor has been identified as being the main driver behind the low use of chemoprophylaxis, a combination of cost and accessibility, together with experiences of side effects from previous drug use, and the cheap and readily available treatments where malaria is endemic appear to contribute to a perception among some VFRs that the risks posed by malaria are outweighed by the drawback of chemoprophylaxis use [12].

### Limitations of the current strategic approach

The mismatch between the threat of malaria as detailed in the current UK malaria prevention guidelines and the experiences of VFRs, particularly in their country of origin, is potentially contributing to low compliance to national policy recommendations. The epidemiological data suggest that the current strategy for addressing this gap, focusing on A (raising awareness) to increase B (bite prevention) and C (use of chemoprophylaxis) has had little impact on the rates of imported malaria among the VFR population over the past ten years. The ABCD approach is based primarily on a knowledge deficit hypothesis and the assumption that a didactic knowledge transfer approach, delivering ‘correct’ and ‘appropriately tailored’ information to VFRs though primary care providers is the most effective way of changing perceptions and practices. While it may be important to increase awareness among VFRs of the potentially fatal consequences of malaria, a key limitation of this approach is, as many studies have demonstrated, that there is no direct link between knowledge and behaviour since health behaviours are grounded in complex socio-ecological context where there are frequently structural and social barriers constraining actions [25]. In particular, several studies have shown that there is no direct association between perceived risk of malaria and use of chemoprophylaxis and compliance with national recommended preventive strategies [8,14,21]. Furthermore, data from a recent small qualitative study among VFRs in London, most of whom were first-generation UK residents, suggests that these participants tended to be dubious about the management of malaria within the NHS and were unlikely to view UK primary care providers as trusted sources of malaria information [12,17]. Two conceptual frameworks have recently been developed to describe the complex socio-ecological context within which VFRs make their malaria decisions [12,14] and both identify key structural and social factors that are likely to influence VFR malaria behaviours and affect their willingness and ability to comply with the current ABCD strategy. That is, as the epidemiological data demonstrate, attempting to raise awareness of the dangers of malaria is an ineffective strategy for malaria prevention among VFRs.

The epidemiological and qualitative data presented in this paper suggest that current policy does not account for the epidemiological and social context of VFR malaria. In order to develop more effective strategies to reduce the burden of malaria among VFRs, incorporating this evidence may be helpful. Additional epidemiological data on the effect of repeated travel to malaria transmission areas on the frequency and severity of clinical episodes among VFRs is needed.

In the meantime, should the current malaria prevention guidelines for VFRs be amended? Can alternative strategies be created that take a different approach? Should a separate strategy be developed for VFR travellers, and if so, how is a VFR defined? Defining a VFR is not straightforward and there is no international consensus on a definition [26].

### Potential solutions? Next steps and new strategies

To address these questions alternative strategies might be considered for the management of the malaria burden among VFRs. One alternative may be changing the role of VFR malaria management through the use of standby emergency treatment (SBET) of malaria, an approach which has been already been adopted by European policy makers for travellers to low-risk, malaria-endemic regions [27]. However, there may be pitfalls and risks in withdrawing prophylaxis and relying on self-treatment when travel is to high-transmission countries. For example, a recent French study where 74% of the participants were immigrants, described the dangers of self-medication for malaria, with self-treatment identified as an important risk factor for travellers developing severe disease (OR 2.9 CI 1.6-5.6) [28]. The consequences associated with self-management include: inappropriate management of non-malarial fevers with an anti-malarial; use of poor quality (counterfeit) drug or sub-optimal dosing when purchasing medicine from street sellers; the potential rapid deterioration of patients with falciparum malaria; and, lack of access to medical supervision should the clinical condition not improve [29]. On the other hand, no published studies exist on the effectiveness of self-treatment among VFRs and, rather than ignoring the fact that these practices occur [12,14], perhaps consideration might be given to how such practices may be refined to encourage safe and effective self-management in this risk group.

An alternative strategy might include the provision of rapid diagnostic testing and a recognized treatment regimen within community-based medical/healthcare settings. If such a policy were to be implemented it would need to be underpinned by effective support structures. Such a strategy may realistic because of the demographics and epidemiology of imported malaria in the UK. Around 88% of all imported *P. falciparum* malaria in 2008, was reported in London [17], where immigrant communities are grouped in boroughs often by country of origin [30] and served by local health services. This ‘clustering’ would facilitate geographically targeted training of community-based providers on the recognition, diagnosis and treatment of malaria and targeted distribution of rapid diagnostic kits and artemisinin combination drugs to community based services in ‘high risk’ areas, particularly specific boroughs in London. With such facilities in place, community-based diagnosis in general-practitioner (GP) surgeries, urgent and walk-in care centres and local accident and emergency (A&E) departments could be set up to provide rapid diagnostics [31] closer to, and more accessible for the malaria patient. Ambulatory treatment of imported malaria can be managed, in similar settings with excellent outcomes, at home, without the need for inpatient observation, where individuals are designated at low risk of complications [31-35].

If consideration were to be given to implementing guidelines specific to VFRs, then there remains the question of whether two different sets of guidelines can exist in parallel for travellers to the same region who differ by ethnicity and reason for travel? Having different preventative policies for different risk groups has a number of potential consequences. Firstly, health professionals would need clear guidance on how to decide which policy suits their traveller. They would need a precise definition of who constitutes a VFR, the journey risk and, most importantly, an understanding of the values and beliefs of the traveller. Secondly, there are cost implications: decisions would have to be made as to who would bear the cost and, if it were the traveller, understanding the influence of costs of standby treatment and diagnostic kits on uptake. Thirdly, there may be legal implications for the prescriber (GP, pharmacist, practice nurse) of self-treatment. Finally, the most challenging is the situation where a non-VFR traveller, such as an expatriate or business traveller, prefers standby treatment rather than taking chemoprophylaxis. How should a health professional deal with such a request? In a Dutch study of 604 malaria patients, the majority being VFRs, full compliance to chemoprophylaxis was associated with significantly lowered odds of developing severe malaria [20]. A policy recommending no prophylaxis therefore raises the potential of more frequent fatal malaria cases. The ‘easy option’ of standby treatment could be a poor and dangerous alternative for tourists, expatriates and similar groups, but could prove difficult not to provide if requested by travellers.

To decide on whether a separate policy might reduce malaria in VFR travellers, more detailed information is required on: the likely cost of implementing self-diagnosis and treatment; the acceptability of diagnosis and treatment within primary or acute/emergency care settings; user acceptability of this radical alternative to chemoprophylaxis; and, the acceptability to health care providers of having to deal with two policies, one for VFRs and one for others.

Potential measurable outcomes that could be used to monitor the effectiveness should parallel guidance on malaria prevention be implemented to VFRs include:

I. A change in the proportion and/or number of VFR malaria cases with no increase in deaths;
II. An increase in the number of non-VFR malaria cases and an increase in deaths.

## Conclusion

The epidemiological evidence suggests that the current guidelines for malaria prevention in the UK are not working effectively for a specific group of travellers, namely VFRs. The reasons for this are several and complex but are influenced by the health beliefs of VFRs and the structural, social, environmental, and economic context within which malaria prevention decisions are made. The current guidelines focus on trying to change awareness of risk using behaviour change communication strategies with little attention to the lived experiences of VFRs and the socio-ecological context of their decision-making. The reviews’ findings argues for decreasing the burden of malaria among VFRs, through a different strategy is required. Altering VFR behaviour and reducing the malaria burden requires more than a didactic knowledge transfer approach as is currently advocated and alternative policy should consider a focus on solving practical issues, including self-management of malaria, early diagnosis and rapid treatment through primary and urgent care centres and easy access to effective malaria treatments. If new guidelines are introduced, research will be critical to ensure changes are carefully evaluated and their impact measured to confirm improvement and safety.

## Abbreviations

VFRs: Visiting friends and relatives
SBET: Standby emergency-treatment
SSA: Sub-Saharan Africa
PHE: Public Health England
ACMP: The Advisory Committee on Malaria Prevention
MRL: Malaria Reference Laboratory
ABCD: Awareness, bite avoidance, chemoprophylaxis, early diagnosis
NHS: National Health Service
A&E: Accident and Emergency Dept.
GP: General Practitioner
